# Application of Mesoporous Silicas for Adsorption of Organic and Inorganic Pollutants from Rainwater

**DOI:** 10.3390/ma17122917

**Published:** 2024-06-14

**Authors:** Anna Marszałek, Ewa Puszczało, Katarzyna Szymańska, Marek Sroka, Edyta Kudlek, Agnieszka Generowicz

**Affiliations:** 1 Department of Water and Wastewater Engineering, Faculty of Energy and Environmental Engineering, University of Technology, 44-100 Gliwice, Poland; anna.marszalek@polsl.pl (A.M.); edyta.kudlek@polsl.pl (E.K.); 2 Department of Air Protection, Faculty of Energy and Environmental Engineering, Silesian University of Technology, 44-100 Gliwice, Poland; ewa.puszczalo@polsl.pl; 3 Department of Chemical Engineering and Process Design, Faculty of Chemistry, Silesian University of Technology, 44-100 Gliwice, Poland; katarzyna.szymanska@polsl.pl; 4 Department of Engineering Materials and Biomaterials, Faculty of Mechanical Engineering, Silesian University of Technology, 44-100 Gliwice, Poland; marek.sroka@polsl.pl; 5 Department of Environmental Technologies, Cracow University of Technology, 31-155 Kraków, Poland

**Keywords:** adsorption, functionalized silica, rainwater, heavy metals, organic compounds

## Abstract

Precipitation is an important factor that influences the quality of surface water in many regions of the world. The pollution of stormwater runoff from roads and parking lots is an understudied area in water quality research. Therefore, a comprehensive analysis of the physicochemical properties of rainwater flowing from parking lots was carried out, considering heavy metals and organic micropollutants. High concentrations of zinc were observed in rainwater, in addition to alkanes, e.g., tetradecane, hexadecane, octadecane, 2,6,10-trimethyldodecane, 2-methyldodecane; phenolic derivatives, such as 2,6-dimethoxyphenol and 2,4-di-tertbutylphenol; and compounds such as benzothiazole. To remove the contaminants present in rainwater, adsorption using silica carriers of the MCF (Mesostructured Cellular Foams) type was performed. Three groups of modified carriers were prepared, i.e., (1) SH (thiol), (2) NH_2_ (amino), and (3) NH_2_/SH (amine and thiol functional groups). The research problem, which is addressed in the presented article, is concerned with the silica carrier influence of the functional group on the adsorption efficiency of micropollutants. The study included an evaluation of the effects of adsorption dose and time on the efficiency of the contaminant removal process, as well as an analysis of adsorption isotherms and reaction kinetics. The colour adsorption from rainwater was 94–95% for MCF-NH_2_ and MCF-NH_2_/SH. Zinc adsorbance was at a level of 90% for MCF-NH_2_, and for MCF-NH_2_/SH, 52%. Studies have shown the high efficacy (100%) of MCF-NH_2_ in removing organic micropollutants, especially phenolic compounds and benzothiazole. On the other hand, octadecane was the least susceptible to adsorption in each case. It was found that the highest efficiency of removal of organic micropollutants and zinc ions was obtained through the use of functionalized silica NH_2_.

## 1. Introduction

Rainwater is an essential component of the water cycle, playing a key role in maintaining ecosystems and providing water for various uses. However, with increasing urbanization, more and more areas are covered with hard surfaces such as asphalt, increasing the amount of rainwater flowing into the sewer system. Rainwater collects contaminants from hard surfaces, such as car oils, street chemicals, and minerals, before entering the sewer system [1,2]. These substances can be harmful to the aquatic environment and pose a health risk. Rainwater flowing into the sewage system can end up in rivers and lakes, introducing pollutants into them and disrupting natural ecosystem processes. This phenomenon may lead to the deterioration of water quality and a threat to aquatic fauna and flora [3,4]. Sources of pollution from roads and traffic include road surface abrasion (10,000 kg/km per year); tire abrasion (0.12 kg/km per 1000 vehicles per year); brake pad abrasion (15 kg per 106 vehicles per kilometre); organic substances such as fuel, transmission oil, grease, brake fluid, and antifreeze (mainly from droplet losses from vehicles); and corrosion products [5]. Tire abrasion generates contaminants such as rubber, soot, and oxides of heavy metals, including zinc (Zn), lead (Pb), chromium (Cr), copper (Cu), and nickel (Ni). On the other hand, abrasion of brake pads is a particular contributor to the emission of nickel (Ni), chromium (Cr), copper (Cu), and lead (Pb) [5].

As evidenced above, rainwater becomes a carrier of various pollutants that can harm both the environment and human health [6]. These pollutants, divided according to their nature into organic and aromatic compounds, can come from emissions from motor vehicles, the petrochemical industry, and inorganics, including heavy metals. Most of these substances are considered micropollutants due to their low concentrations in water, air, or soil environments. Micropollutants are characterized by the fact that they are present in very small quantities, but can have potentially harmful effects on human health and ecosystems [7]. Their negative impacts on health include, among others, neurodevelopmental defects in children, thyroid disease, bone deformities, and endocrine cancers. Furthermore, these pollutants deteriorate soil conditions, affecting microbes and biogeochemical processes, which can lead to genetic changes in microorganisms [8]. Precipitation is an important factor that influences the quality of surface water in many regions of the world. However, stormwater runoff pollution from roads and parking lots is an understudied area in water quality research. Numerous studies can be found in the literature, most of which have focused on different types of pollutants. In the southwestern United States, atrazine, prometon, simazine, pentachlorophenol, nonylphenol, and PFOA have been detected in rainwater. Average concentrations were significantly higher during the monsoon season than in winter for prometon, simazine, and pentachlorophenol. However, average concentrations of nonylphenol were significantly higher in winter than during the monsoon season [9]. In France, new metals (As, Ti, Sr, and V) and organic substances—such as nonylphenol and octylphenol ethoxylates, some pesticides, and bisphenol A—have been determined in rainwater [10]. However, in rainwater collected from urban areas and agricultural land in northern Thailand, caffeine, 4-nitrophenol, tris(2-butoxyethyl) phosphate, atrazine, phenobucarb, and 2,4-D were determined. In addition, evidence of the presence of acetaminophen, fexofenadine, diphenhydramine, and gabapentin was provided [11]. Extensive studies on surface runoff have been carried out in the centre of Aachen, western Germany, where all polycyclic aromatic hydrocarbons, benzothiazoles, benzotriazoles, and organophosphates were analysed. They are classified as urban and road pollutants. Tris(1-chloro-2-propyl)phosphate (TCPP, 1.6 mg/kg silicone) was also detected, which was believed to have come from car interiors and was found in snow samples near road intersections [12].

However, the concentrations of heavy metals in precipitation waters, both those coming from paved surfaces and those from roofs, are the most widely discussed. For example, the concentration of heavy metals in rainwater from the metropolis and suburbs of Lagos State in Nigeria is as follows: Fe > Zn > Ni > Cu > Cd > Pb. Cd and Pb exceed WHO standards [13]. On the contrary, in Istanbul, metal concentrations in rainwater were found to be higher than those reported by other researchers around the world. The solubility of toxic metals was found to follow the order of Cd > Cu > V > Zn > Ni > Pb > Cr [14]. On a subtropical island off the coast of northern Taiwan, Cu, Zn, Cd, and Pb were detected in rainwater, and the authors confirmed that they come primarily from anthropogenic sources, related to industrial combustion and the emissions from local road traffic [15]. In Lucknow, India, sufficiently high concentrations of chromium, cadmium, and lead have been found to exceed the permissible limit established by the WHO, which can cause various adverse health effects [16]. All these data emphasize that the place (agricultural or industrial areas), the season, the type of surface, and air pollution play a significant role in the type and amount of pollutants present. It is worth noting that the presence of micropollutants highlights the need for additional treatment processes to neutralize potentially negative effects on the environment [17,18]. An adsorption process can be an effective solution due to its ability to efficiently remove contaminants from water [19]. In addition, the process can be adjusted by selecting the appropriate adsorption carrier and optimizing the process conditions—such as adsorbent dose, contact time, and temperature—to achieve high contaminant removal efficiency. Additionally, due to its relatively simple implementation and low operating costs, the adsorption process is often the preferred method of stormwater treatment compared to other technologies [20]. MCF silica carriers were introduced to remove contaminants. These cell foams, made of silica and with a mesoporous structure, are characterized by high adsorption efficiency. Functionalized silica carriers, on the other hand, are materials that have been chemically modified to obtain specific properties, such as chemical reactivity or adsorption capacity aimed at specific substances [21]. Silica carriers are commonly used as adsorption materials to remove a variety of contaminants from a variety of environments, including surface water, groundwater, and gases [22,23,24]. They are minerals composed mainly of silica (silicon dioxide, SiO_2_) and can have different forms, such as colloidal silica, activated silica, or functionalized silica [25]. Silica can effectively absorb a variety of contaminants, including heavy metals [26,27,28], organic compounds [29], dyes [30,31], and other harmful substances [32]. The silica used in this study has been further functionalized to improve its properties. The functionalization process involves chemical modification of the silica surface by introducing specific functional groups onto it [33]. This modification is intended to improve the ability of silica to adsorb specific contaminants. For example, by adding amino groups, the affinity of silica for the adsorption of dyes or heavy metals can be increased [34,35]. Three groups of modified carriers were prepared—(1) SH (thiol), (2) NH_2_ (amino), and (3) NH_2_/SH (amine and thiol functional groups). The research problem, which is addressed in the presented article, is concerned with the silica carrier influence of the functional group on the adsorption efficiency of micropollutants. The modification of MCF using two groups at the same time was aimed at increasing the efficiency of removing both organic and inorganic compounds from water. This is an important aspect, as choosing the right carrier can be crucial for the efficiency of the rainwater removal process. An important element of this article is the comprehensive analysis of the physicochemical properties of rainwater flowing from parking lots, including heavy metals and organic micropollutants. The study also included an evaluation of the effects of adsorption dose and timing on the efficiency of the contaminant removal process, as well as an analysis of adsorption isotherms and reaction kinetics, allowing us to better understand the mechanisms involved in the adsorption process. An additional novelty in the presented article is the fact that the study of the adsorption of micropollutants was carried out on actual rainwater. Until now, many studies on adsorption processes have used laboratory models or artificially prepared contaminant solutions, which can lead to some simplifications and inaccurate representation of real environmental conditions. Real rainwater can contain a variety of chemicals, microorganisms, and other components that can affect the adsorption process in ways that are difficult to predict in laboratory models. The results obtained from such studies may be more reliable and useful for engineering practice, as they reflect the conditions under which adsorption processes are to be used for rainwater treatment in real terms.

## 2. Materials and Methods

**Figure 1:**
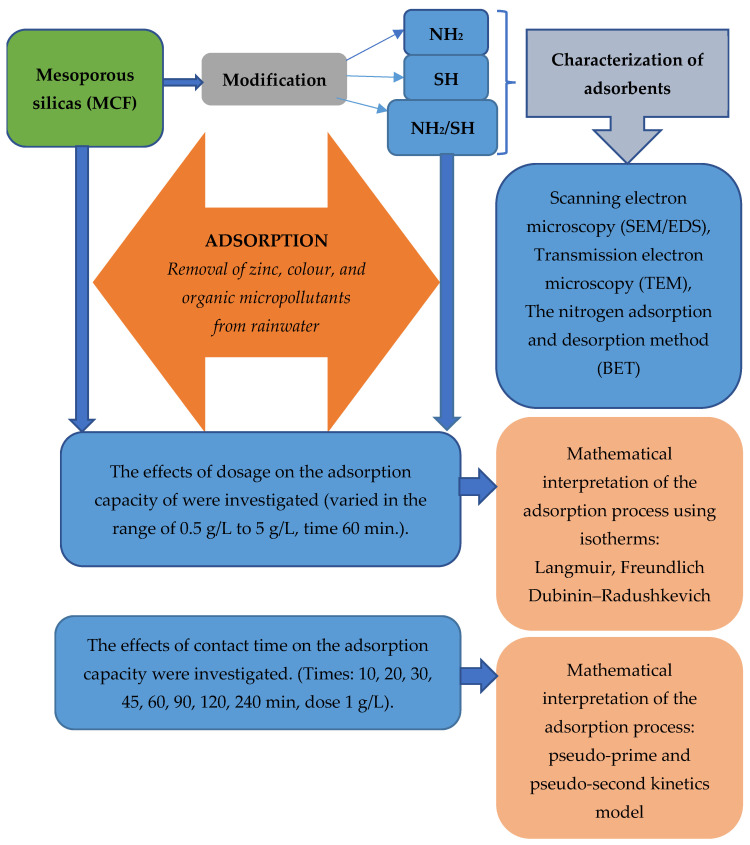
Stages of the testing of mesoporous silicas, including the assessment of their physicochemical properties and the characteristics of the adsorption process.

In this section, we will discuss how we collected data, performed analyses, and interpreted the results. To simplify the understanding of this process, we have created a diagram that presents the next steps and stages of our research work. In the description below, we explain the different parts of the diagram and what they mean for a full understanding of our research methodology. Figure 1 schematically shows the different stages of the research.

### 2.1. The Subject of the Study

**Figure 2:**
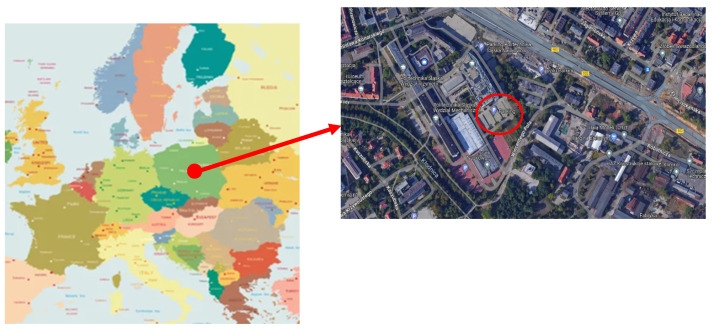
Location of the rainwater collection site used for the research.

The subject of the study was rainwater from the manhole parking lot of the Silesian University of Technology, located on the campus in Gliwice, Silesian Voivodeship, Poland. Near the place from which the water was collected is the Diameter Road 902. The exact location of the sample collection site is shown in Figure 2. Rainwater was collected in March 2023.

### 2.2. Analysis of Organic Micropollutants

The chromatographic analysis of the extracts was performed using equipment from Agilent Technologies (Santa Clara, CA, USA). The use of the analytical tool was consistent with the methodology described in a previous article [36]. Compounds were identified by comparing their mass spectra with data from the NIST v17 database. A compound was considered identified if the precision of matching its spectrum to the proposed spectrum in the database (expressed as similarity in %) was greater than or equal to 70%.

### 2.3. Analysis of Physicochemical Parameters

The efficiency was evaluated by monitoring typical quality parameters (colour, COD, TOC, cooper, nickel, zinc, lead, nitrate nitrogen, phosphate phosphorus, conductivity, pH).

### 2.4. Synthesis and Modification of MCF Materials

The preparation of MCF was performed as described in [37,38]. In a typical procedure, Pluronic P123 (4 g) was dissolved in 1.6 M HCl (75 mL) at room temperature. Then 1,3,5-trimethylbenzene (5.8 mL) and NH_4_F (0.023 g) were added by vigorous stirring and the mixture was heated to 40 °C. Following 1 h stirring, TEOS was added (4.7 mL), whereupon the mixture was stirred for 1 h and then stored at 40 °C for 20 h, then at 100 °C for 24 h. After being cooled to room temperature, the precipitate was filtered, dried at room temperature for 4 days, and calcined at 500 °C for 8 h.

Before grafting, MCFs were contacted with water vapour for 5 h and then dried at 200 °C for 2 h. Functional groups were grafted onto the silica surface by reacting the appropriate amounts of organosilanes (3-aminopropyltrimethoxysilane (-NH_2_) and/or 3-mercaptopropyltrimetoxysilane (-SH)) in toluene under reflux (24 h, 80 °C), with the silanols present on the silica surface to obtain the load of the functional moiety of approximately 1.5 mmol/g of silica. In particular, 50 mL of the solution containing 3-aminopropyltrimethoxysilane (0.269 g for MCF-NH_2_ or 0.135 g for MCF-NH_2_/SH) and/or 3-mercaptopropyltrimetoxysilane (0.295 g MCF-SH or 0.147 g MCF-NH_2_/SH) was stirred under reflux with 1 g of MCF for 24 h, after which the solvent was removed by filtration. Unmodified silicas were designated as MCF, those modified with amine groups as MCF-NH_2_, those with thiol groups as MCF-SH, and those containing both groups as MCF-NH_2_/SH.

### 2.5. Adsorption Process

The influence of contact time and sorbent dosage on the adsorption capacity of zinc and colour from rainwater was investigated. Additionally, an experiment on the adsorption of organic micropollutants was conducted using a fixed adsorbent dosage (1 g/L) and a selected adsorption time (120 min). The adsorption process took place in 100-millilitre glass flasks containing 100 millilitres of the test solution, at room temperature (20 ± 2 °C), in an incubator with a shaker rotating at 300 revolutions per minute. Kinetic experiments were conducted as follows: 1 g/L of sorbent was added to 100 millilitres of feeding water in a glass flask, which was then shaken at 300 revolutions per minute for specific time intervals: 10, 20, 30, 45, 60, 90, 120, 240 min. The pH of the solution was kept constant using a 0.1 mol/L HNO_3_/NaOH solution. Samples of the adsorbent were separated from the solution at specified time intervals for further analysis. Similarly, an equilibrium adsorption experiment was conducted. The mass of sorbents varied from 0.5 g/L to 5 g/L for 120 min.

To determine the adsorption kinetics, the experimental data were analysed using pseudo-first-order and pseudo-second-order kinetic models, which are given in Equations (1) and (2), respectively [39]:

(1) $$\ln\frac{qe-qt}{qe}=-K_{1}*t$$

(2) $$\frac{t}{qt}=\frac{1}{K_{2}*{ge}^{2}}+\frac{t}{ge}$$

where q = quantity of adsorbed (mg/g) and t = time, the slope of the linear plot of ln(qe − qt) against t gives –K_1_, where K_2_ can be estimated from the linear plot of t/q_t_ vs. t.

The amount adsorbed was calculated from Equation (3) [40],

(3) $$Q_{e}=\frac{\left(C_{0}-C_{e}\right)*V}{m}$$

where Q_e_ (mg/g) is the amount adsorbed, C_0_ and C_e_ (mg/L) are the initial and equilibrium concentrations of the adsorbates, respectively, m (g) is the mass of the adsorbent, and V (L) is the volume of the feed water.

Two common adsorption isotherm models were used to fit the experimental data in this study, including the Langmuir model, the Freundlich model, and the Dubinin–Radushkevich model. Each model is briefly described below.

The Langmuir model assumes monolayer adsorption, where molecules interact only with the surface of the sorbent. The Langmuir isotherm is represented by Equation (4) [40],

(4) $$\frac{1}{Q_{e}}=\frac{1}{Q_{m}K_{L}}\cdot \frac{1}{C_{e}}+\frac{1}{Q_{m}}$$

where Q_m_ (mg/g) is the maximum adsorption capacity and K_L_ (L/mg) is the Langmuir fitting parameter.

The Freundlich model is empirical and adequately describes adsorption on heterogeneous surface energy systems. This model has significant importance in chemisorption and some cases of physisorption, and it can be written as shown in Equation (5) [40],

(5) $$\log q_{e}=logK_{F}+\frac{1}{n}logC_{e}$$

where K_F_ ((mg/g)(L/mg)^n^) is the Freundlich adsorption coefficient and n is the number describing surface heterogeneity and sorption intensity.

The Dubinin–Radushkevich model is based on the postulation that the mechanism for adsorption in micropores is that of pore-filling rather than layer-by-layer surface coverage. The Dubinin–Radushkevich equation takes the form shown in Equation (6),

(6) $$\ln q_{e}=\ln a_{DR}-E^{-2}\cdot \varepsilon^{2}$$

where a_DR_ (mg/g) is the amount of adsorbate that can be adsorbed in micropores (this can be obtained by plotting lnq_e as a function of ε^2^), E (kJ/mol) is the adsorption energy (which can be read from the slope of that line), and ε is the adsorption potential, which is defined by Equation (7),

(7) $$\varepsilon =R\cdot T\cdot ln\frac{C_{s}}{C_{e}}$$

where R (8.314 J·mol^−1^·K^−1^) is the ideal gas constant, T (K) is the temperature, and C_s (mg/L) is the solubility in water [40].

### 2.6. Characterization of Adsorbents

The characterization of MCF included the following:

- The surface morphologies of the minerals studied were investigated by scanning electron microscopy (SEM/EDS). The scanning electron microscope used for the JSM 6360LA was manufactured by JEOL-Japan (Tokyo, Japan).
- The textures of the materials were also examined using transmission electron microscopy (TEM, Tecnai G2 apparatus operating at 200 kV).
- The structural properties of the adsorbents were determined by measuring the surface areas and pore size distributions using the low-temperature nitrogen adsorption and desorption technique, according to the Brunauer–Emmett–Teller (BET) method.
- The determination of functional groups and vibrational spectra was performed using FTIR spectroscopy.

### 2.7. Statistical Analysis of the Results

The results presented in the figures and tables in this paper are the obtained values of the results of the experiment, calculated using the arithmetic method. The removal degree of the compound ions of the MCF studied was calculated using the following Equation (8):

(8) $$\mathrm{Removal}\;\mathrm{degree}\;(\%)=\frac{C_{0}-C}{C_{0}}\times 100\%$$

where C_0_ is the initial concentration of the compound in the solution (mg/L) and C is the concentration of the compound in the solution after adsorption (mg/L).

The experimental errors are presented as confidence intervals, and error bars are given as standard deviation, σ, according to Equation (9):

(9) $$\sigma =\sqrt{\frac{\sum_{i=1}^{n}{\left(x_{i}-x\right)}^{2}}{n-1}}$$

where x represents the results of a single experiment repetition and n—is the number of repetitions.

## 3. Results and Discussion

### 3.1. Characteristics of MCF

**Figure 3:**
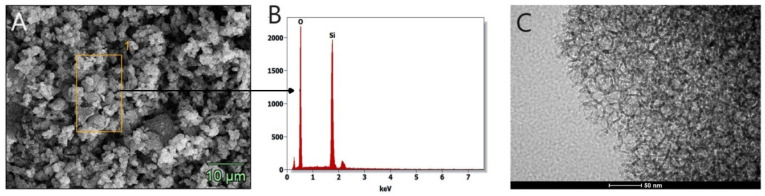
SEM (**A**), EDS analysis (**B**), and TEM (**C**) images of MCF carrier.

Mesoporous cellular foams (MCFs) are characterized by well-defined pores with a uniform size distribution and shape. The pore diameter is about 25 nm and the size of the connecting windows is about 15 nm (Table 1). The SEM image (Figure 3A) shows an extended structure, which affects the size of the specific surface (about 500 m^2^/g, Table 1, obtained using the BET method). The elemental composition of the materials was confirmed by EDS analysis, showing that the unmodified MCF-type support consists exclusively of silicon and oxygen (Figure 3B). The cellular structure of the MCF was confirmed by the TEM image (Figure 3C).

**Figure 4:**
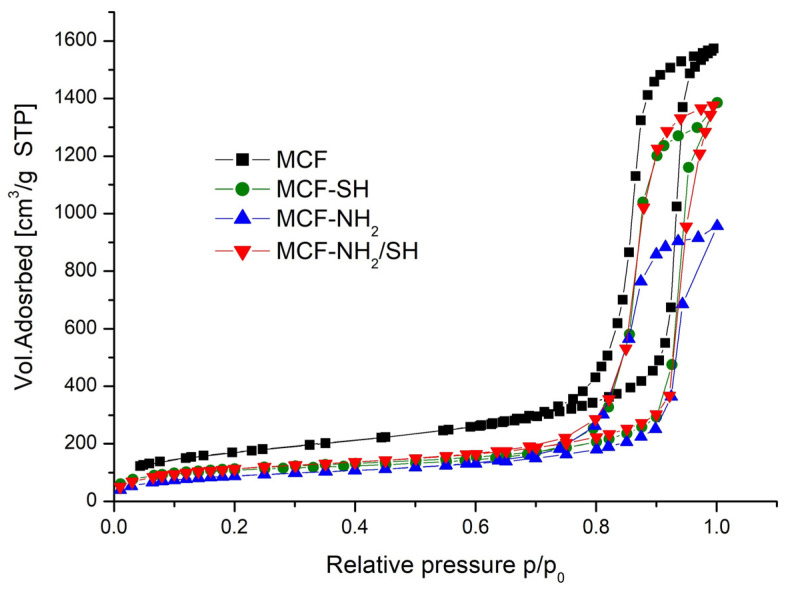
Nitrogen adsorption and desorption isotherms of silica mesoporous cell foam (−196 °C) before and after its functionalization.

Figure 4 shows the nitrogen adsorption and desorption isotherms of mesoporous cellular foam (MCF) at −196 °C before and after functionalization. These are classified as Type IV isotherms, characterized by a steep H1-type hysteresis in the medium to high relative pressure range typical of mesoporous materials. H1 type hysteresis loops are obtained when the capillaries are shaped as bilaterally open, regular, and irregular cylinders or prisms [41,42].

Table 2 shows the texture parameters of cellular foams (MCFs) before and after functionalization with organosilanes. As a result of functionalization, there was a reduction in the volume of adsorbed nitrogen (pore volume), a reduction in the diameters of the pores and the windows connecting them, and a reduction in the specific surface area. This is confirmed by other studies [43,44]. These changes are proportional to the number and size of organic groups introduced.

**Figure 5:**
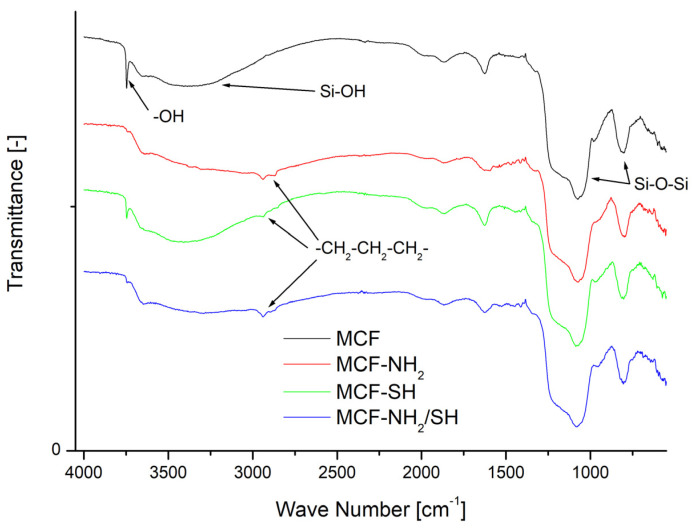
FTIR spectra of the pristine MCF after functionalization.

To confirm the presence of organic functional groups on the surfaces of silica carriers, infrared spectra were analysed (Figure 5).

Figure 5 shows the FTIR spectra of the MCF material in its pristine form (MCF) and after modification (MCF-NH_2_, MCF-SH, MCF-NH_2_/SH). The unmodified silica featured a broad band between 3200 and 3600 cm^−1^ that could be attributed to Si-OH stretching vibrations; additionally, a sharp and intense band at 3740 cm^−1^ could be unambiguously assigned to the OH symmetric stretching vibration of isolated silanol groups [43]. The adsorption bands around 1000–1250 cm^−1^ and 800 cm^−1^ are attributed to asymmetric and symmetric stretching vibrations of the Si-O-Si framework [45]. Appreciable changes in all of the spectra were observed after modification of the MCF support with organic moieties. First, the intensity of the band at 3740 cm^−1^ was markedly reduced as a result of the incorporation of organic groups. The presence of –NH_2_ and –SH groups were identified by the methylene stretching bands of the propyl chain in the region of 2850 to 2950 cm^−1^, seen in the FTIR spectra of MCF-NH_2_, MCF-SH, and MCF-NH_2_/SH, and their deformation bands at 1410 to 1455 cm^−1^. The N–H absorption bands overlapped with O–H bands at 3200–3600 cm^−1^ [46].

### 3.2. Characteristics of Rainwater

**Figure 6:**
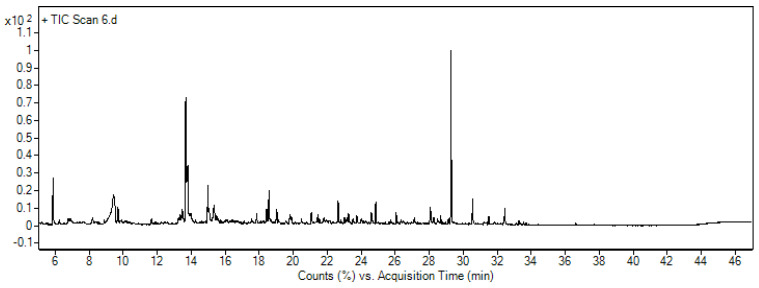
A chromatogram obtained for rainwater before the treatment process.

The general characteristics of rainwater and the contents of their organic and inorganic compounds are presented below. Table 3 and Table 4 present the physicochemical parameters and the identification of the organic compounds in rainwater, respectively. Figure 6 shows a selected chromatogram from the analysis of the actual rainwater flowing from the parking lot.

Based on the results of the physicochemical analysis, it was found that almost all parameters of the tested rainwater did not exceed the standards specified in the Regulation of the Minister of Maritime Economy and Inland Navigation [47]. This regulation establishes the permissible values of substances particularly harmful to the aquatic environment and conditions to be met when discharging wastewater into water or the ground, as well as when discharging rainwater or snowmelt into water or water facilities. In the analysed water, of all the parameters tested, only the concentration of zinc exceeded the standards (2 mg/L) specified in the Regulation [47]. This contaminant may be present in the water due to abrasion from car tires [48,49]. Other researchers have also detected zinc in water from an urbanised catchment of a rainwater collector in Gdansk [50]. These data are also corroborated by researchers who analysed rainwater in Berlin [49].

The tested rainwater also contained alkanes, both simple (e.g., tetradecane, hexadecane, octadecane) and branched (2,6,10-trimethyldodecane, 2-methyldodecane), as well as halogenated alkane derivatives (1-chloroctadecane), phenolic derivatives, and benzotriazole. Higher alkanes are used in the production of fuels and lubricants [51]. Benzothiazole (BT), on the other hand, is an organic chemical and is a heterocyclic compound containing a benzene ring linked to nitrogen and a sulphur atom. There are several potential sources of benzothiazole in rainwater from the street—it can come from vehicle exhaust emissions, especially motor oils; fuels; and materials that wear out during vehicle operation (such as tires or brake pads) [52].

If there are industrial plants in the vicinity of the road that use benzothiazole or benzothiazole derivatives in their production processes, this substance can leach into stormwater [53]. Pollutants generated by human activities, such as vehicle emissions and industrial activities, are contained in road dust. This dust can be released into the atmosphere by re-emitting particles, which contributes to air pollution. Then, during the rainy season, this dust can be flushed into water bodies, leading to pollution of the aquatic environment [54].

Rainwater quality analysis is a very useful tool for understanding air quality and the origin of the pollutants it contains. Contaminants present in rainwater can be chemical, physical, or biological. In addition to atmospheric pollutants, there are a number of pollutants on the road that come from vehicles, such as metals and other materials resulting from the degradation of tyres and other parts, as well as by-products of internal combustion engines and corrosion processes [55]. In Poland, the quality of rainwater is regulated by the Regulation of the Minister of Maritime Economy and Inland Navigation, Journal of Laws 2019, item 1311. In the European Union, on the other hand, the main directives for the management of urban stormwater runoff are the EU Water Framework Directive (WFD) of 2000 and the Environmental Quality Standards Directive (EQSD) of 2008 [56]. In the USA, regulations have been implemented mainly on the basis of arrangements issued by the Environmental Protection Agency (EPA) since 1972 [57]. On the basis of the literature, it has been concluded that rainwater is a service of local responsibility—regulations concerning rainwater are dispersed at all levels of the administrative organization of the state and in all localities [57]. It was found that the general lack of clear guidelines and regulations for the management of stormwater quality and its potential risks to public health may hinder the implementation of stormwater programmes [58]. On the other hand, it is believed that identifying sources of pollution in catchments is crucial for accurately predicting rainwater quality.

### 3.3. Efficiency of Adsorption of Micropollutants Using Silica

**Figure 7:**
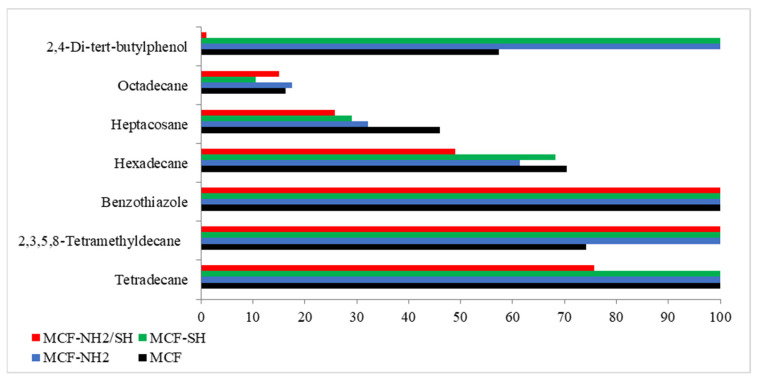
Degrees of removal of micropollutants for each adsorbent used.

Figure 7 shows the degree of removal of each organic micropollutant through the adsorption process. The degrees of removal were determined based on the peak areas of the compounds present in rainwater before and after the adsorption process.

There are a limited number of studies in the scientific literature on the adsorption of organic micropollutants using silica, as well as studies using real aqueous solutions. Our analysis showed that all silica tested achieved 100% benzothiazole removal. The highest efficiency of removal of all considered organic micropollutants was found for the silica MCF-NH_2_. Studies by Hafezian et al. [59] confirm the efficacy of the NH_2_ group for sulforaphane adsorption. It was noted that in the case of phenolic impurities, the thiol group showed the same adsorption efficiency as the amino group. Octadecan was the least susceptible to adsorption. Alkanes typically do not adsorb strongly on adsorption surfaces compared to more polar compounds such as alkenes, alcohols, or carboxylic acids. This is due to their low polarity and weak intermolecular interactions [60]. In addition, a number of various other chemical compounds result in a lack of available adsorption sites [61].

Batch adsorption studies show that the adsorption capacity of contaminants in functionalized silica is greater than in pure MCF. It was also found that functionalization of MCF with two thiol and amine groups reduced the efficiency of adsorption.

### 3.4. Effects of Process Time and Adsorbent Dose on the Efficiency of Zinc and Colour Adsorption Efficiency from Rainwater

**Figure 8:**
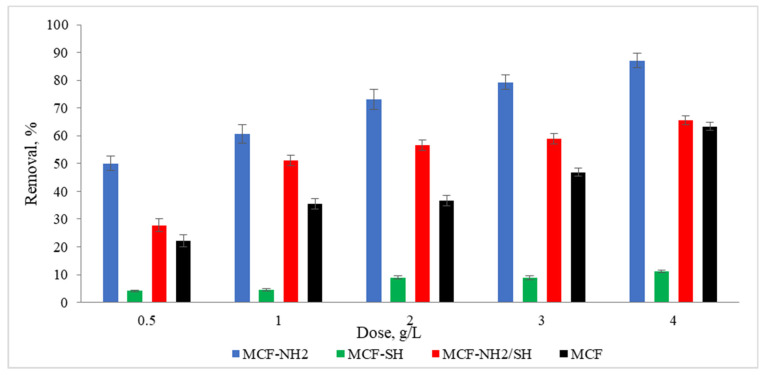
Effect of adsorbent dose on the concentration of zinc in rainwater (C_0_ = 4.5 mg/L, time of processes = 120 min, pH rainwater = 6.7).

**Figure 9:**
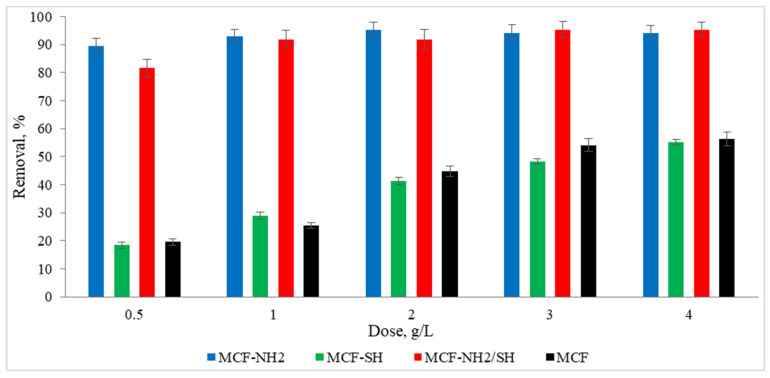
Effect of adsorbent dose on colour in rainwater (C_0_ = 87 mg/L, time of processes = 120 min, pH rainwater = 6.7).

To meet the water regulatory standards imposed by governments and other regulators, many advances are being made in improving the water purification process. One such advancement involves functionalized mesoporous silica. This study proved that the large surface areas of mesoporous materials and the possibility of their functionalization with different functional groups make these materials ideal for use in the adsorption area, for example, for the removal of heavy metals and organic pollutants from rainwater. Studies were carried out on the effect of the dose of the adsorbent on zinc and colour adsorption from actual precipitation water (Figure 8 and Figure 9). It was found that the functionalization of MCF with an amino group gave positive results, as the concentration of zinc ions in rainwater was 49% lower than in the case of pure MCF used at a dose of 1.0 g/L. Additionally, an increase in dye removal of 53% was noted. In the context of the adsorption of heavy metals, in this case zinc, a significant performance advantage of amino group-functionalized MCF over thiol group-functionalized MCF was found. A high efficiency of zinc adsorption by amine-functionalized silica carriers was also achieved by Kazuma Nakanishi et al. [62]. In terms of colour removal, MCF-NH_2_ also achieved the highest efficiency. However, the colour adsorption capacities of MCF-SH and MCF-NH_2_/SH were higher than their zinc adsorption capacities. It is assumed that silica carriers with a thiol group will remove organic compounds or colour more effectively, and carriers with an amino group will be dedicated to the adsorption of heavy metals from rainwater. Therefore, we decided to combine both functional groups with a silica carrier. Some authors suggest that hydrogen bonds moderated by a water molecule may form between the groups that make up the colour of water and amino groups [63].

**Figure 10:**
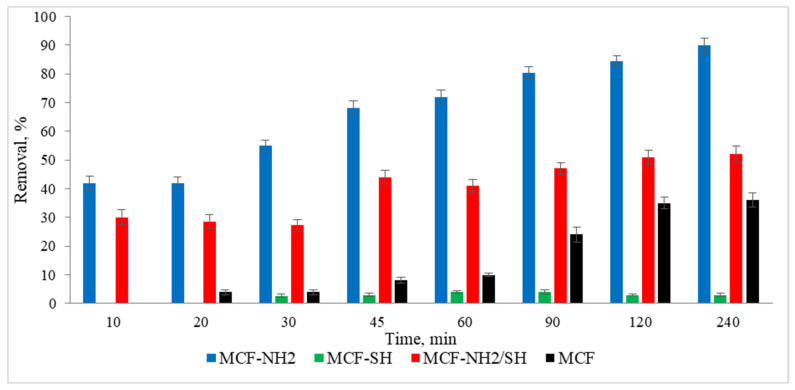
Effect of processing time on the effectiveness of the removal of colour from rainwater (C_0_ = 5 mg/L, dose of adsorbents = 1 g/L, pH rainwater = 6.7).

**Figure 11:**
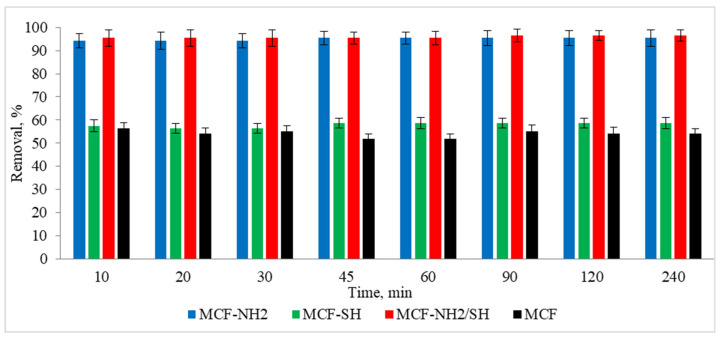
Effect of processing time on the effectiveness of the removal of zinc from rainwater (C_0_ = 87 mg/L, dose of adsorbents = 1 g/L, pH rainwater = 6.7).

The next stage of the research was to determine the effect of the process duration on the adsorption efficiency. The tests were carried out at a constant room temperature. A real rainwater solution was used, in which the zinc concentration was 5 mg/L and the colour was 87 mg Pt/L. Studies were carried out using a dose of 2 g/L and different adsorption times in the range of 10–240 min. The results are shown in Figure 10 and Figure 11.

In the case of colour, rapid and consistent adsorption was recorded after just 10 min of the process. The efficiency was 94–95% for MCF-NH_2_ and MCF-NH_2_/SH, while for MCF-SH it was 56–58%, and for pure MCF it was 51–54%. On the other hand, zinc adsorption increased with the duration of the process and for 240 min it was removed at a level of 90% for MCF-NH_2_; 52% for MCF-NH_2_/SH; for MCF-SH, only 3%; and for pure MCF, 36%. The effectiveness of silica carriers functionalized with amine groups has been confirmed by numerous studies—for example, they were used for the adsorption of chromium from water [64], as well as phytic substances [65]; copper, nickel, zinc, cadmium, and lead [66]; uranium (VI) [67]; fufic acids [68]; nitrates [69]; and dyes [70]. Other applications of silica were determined by Flores et al., who conducted research on the use of various types of mesoporous silica and showed that among the materials studied, mesoporous silica nanoparticles (MSNPs) turned out to be a highly efficient and versatile adsorbent, showing exceptional efficiency in the removal of both metal ions (iron, nickium, and copper) and organic dyes (MB and MG) [71]. In addition, magnetic mesoporous silica (NSMSiO_2_) showed a high adsorption capacity for humic substances and cationic dyes [72]. Yet another study has shown that hexagonal (MCM-41) mesoporous materials can be highly efficient in adsorbing trichloroethylene and tetrachloroethylene from water [73]. Wang et al. compared toluene adsorption by macroporous and mesoporous silica and proved that mesoporous silica exhibited a toluene adsorption capacity much higher than macroporous silica due to its very large surface area [74]. In summary, mesoporous silica materials, especially those functionalized with specific groups such as amines, exhibit excellent adsorption properties for various contaminants, including metals, dyes, and organic compounds. Their large surface areas and functionalization potential make them versatile and effective for environmental cleaning applications.

### 3.5. Zinc Adsorption and Colour Isotherms and Kinetics for MCF-NH_2_

**Figure 12:**
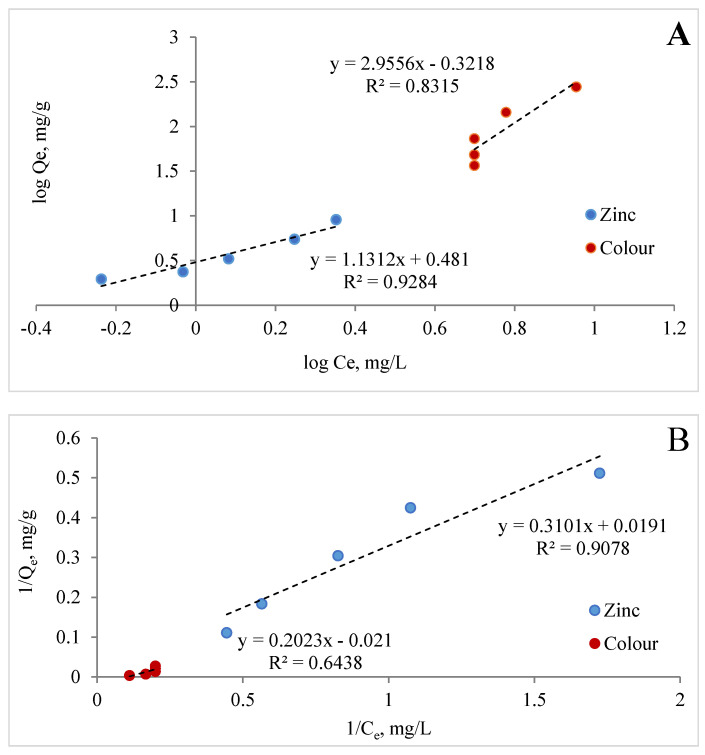

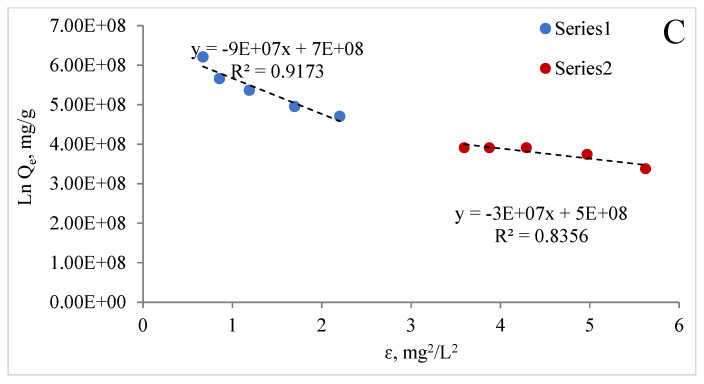
Freundlich (**A**), Langmuir (**B**), and Dubinin-Radushkevich (**C**) isotherms of zinc and colour for MCF-NH_2_.

Adsorption isotherms are very important for describing the interaction between the adsorbate and the adsorbent at equilibrium. Figure 12A–C show the Freundlich, Langmuir, and Dubinin–Radushkevich isotherms of Zn (II) and colour.

It is known that the magnitude of the apparent adsorption energy E is useful in estimating the type of adsorption, and if this value is less than 8 kJ/mol the type of adsorption can be explained by the physical adsorption; when E is between 8 and 16 kJ/mol, the type of adsorption can be explained by ion exchange; and when the value is over 16 kJ/mol, the adsorption type can be explained by stronger chemical adsorption than ion exchange [75]. The energy value obtained from Dubinin–Raduszkiewicz is less than 8 kJ/mol, suggesting the physical adsorption of zinc and colour by MCF-NH_2_. It was found that the correlation coefficients of the Langmuir graph model are 0.91 and 0.64, respectively; the Freundlich graph model is 0.93 and 0.34; and the Dubinin–Raduszkiewicz correlation coefficients are 0.917 and 0.836, respectively, for zinc and colour. Table 5 presents the parameters estimated from linear isotherm data plots. The data show that the Langmuir isotherm model provides a much better fit than the Freundlich isotherm model for colour adsorption. However, in the case of the adsorption of zinc and colour described by the Freundlich model, the values of n were greater than 0 and less than 1, which also suggests favourable adsorption [76,77]. Thus, the adsorption of zinc ions and colour by MCF-NH_2_ can be consistent with the Langmuir and Freundlich isotherm models. In other words, the surface of MCF-NH_2_ can typically be homogeneous, with metal ions adsorbing through surface amino groups. In addition, the R^2^ calculated from all models and the E < 8 kJ/mol suggest that adsorption may be compatible with the pore-filling mechanism. On the other hand, Hongjie WANG et al., in their studies on the functionalization of silica carriers and their application to copper adsorption, found that adsorption by binding metal to organic functional groups may be involved [78]. The assumption that types of functional groups have a significant effect on the removal capacity and selectivity of target heavy metal ions is supported by a review by Peng Zhang et al. [79]. These functional groups on the surface of ordered mesoporous materials tend to form stable coordination compounds with heavy metals, so heavy metal ions can be removed from the aqueous solution. In summary, adsorption involves the mechanisms of both chemisorption and pore-filling.

**Figure 13:**
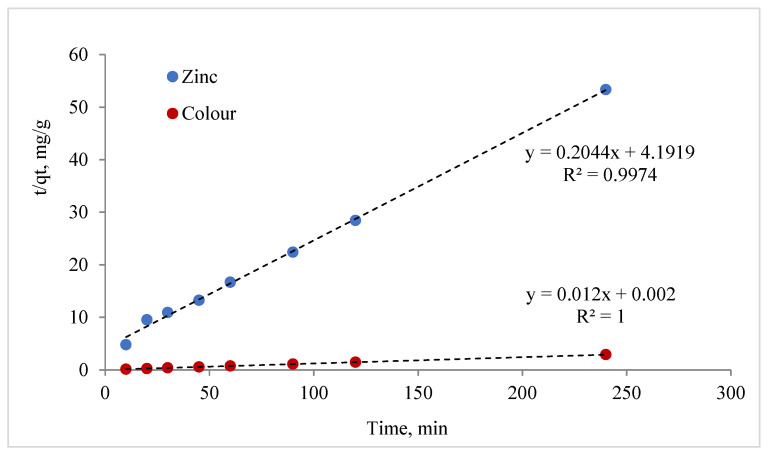
The pseudo-second-order kinetic model for the adsorption of zinc and colour by MCF-NH_2_.

In the present study, a batch experiment investigated the effect of contact time on the interactions between MCF particles and zinc ions and rainwater colour. The maximum adsorption capacities Qe, K_1_, and K_2_ and the correlation coefficient R^2^ calculated from the pseudo-first- and second-order models are shown in Table 6. In the case of R^2^, the values of the pseudo-second-order model are larger than in the pseudo-first-order model. This suggests that the adsorption proceeded according to second-order kinetics and that the model used can be used to determine the relevant kinetic parameters. The pseudo-second-order model (Figure 13) describes the zinc and colour adsorption of the samples very well. The adsorption of the colour was faster than that of the zinc ions.

## 4. Conclusions

In light of the obtained results, it is confirmed that the selection of the appropriate functional group should be taken into account during the adsorption process. However, the assumption of equivalent effectiveness of the two functional groups seems to be accurate only in the context of the elimination of the natural colour of rainwater. This colour can be the result of both organic and inorganic pollutants generated by dust and exhaust fumes from vehicles. In addition, the study confirms that the analysis of the adsorption process using real solutions may lead to different conclusions than the analysis of the removal of individual contaminants. Continued research on the use of silica as an adsorbent carrier may contribute to the development of effective and environmentally friendly solutions for the removal of micropollutants from rainwater.

Based on the research carried out, the following conclusions can be drawn:

1. The results of the analysis of rainwater by the applicable Polish Regulation on the quality of rainwater show that the permissible concentration of zinc was exceeded. In addition, various compounds such as alkanes, phenol derivatives, and benzothiazole were found.
2. MCF-NH_2_ was found to be effective in the adsorption of zinc ions and colour.
3. On the other hand, MCF-NH_2_/SH showed high colour removal efficiency, while in the case of zinc removal it was less effective than MCF-NH_2_. Studies confirm the effectiveness of modified MCF in the removal of organic micropollutants, especially phenolic compounds and benzothiazole. However, the alkanes were not adsorbed, probably due to their low polarity and weak intermolecular interactions.
4. It was found that the adsorption of zinc and colour by MCF-NH_2_ can be consistent with the Langmuir and Freundlich isotherm models. Research suggests that functionalization of silica carriers may affect their ability to remove heavy metals. In conclusion, adsorption is affected by the mechanisms of both chemisorption and pore-filling, and functional groups on the surfaces of porous materials form stable compounds with heavy metals.
5. It was found that the adsorption was carried out according to second-order kinetics—the model used can be used to determine the relevant kinetic parameters.

The obtained results confirm the practicality of taking into account the selection of the appropriate functional group in the adsorption process, and that attention should be paid to the limited accuracy of the assumption of equivalent effectiveness of two functional groups. In addition, it is important to note that analysis of the adsorption process using real solutions can provide new insights, suggesting the need for further research in this area. The development of methods for rainwater treatment and the elimination of micropollutants should be treated as a continuous process, requiring constant monitoring and improvement.

This article shows that contaminants found in parking lot water are often similar in many locations. These pollutants come from sources such as tire abrasion and diesel fuel residues. Literature analysis has confirmed that zinc is a common pollutant in road rainwater, which has also been shown in studies conducted in various cities around the world. Pollution concentrations may vary depending on the intensity of rainfall and the type of road surface, along with other factors, such as the proximity of industrial plants.

It was shown that the proposed process and adsorbent effectively removed many pollutants, although there were difficulties with the adsorption of alkanes. Our paper makes an important contribution to the development of this field by suggesting that this solution can be applied in various locations.

## Figures and Tables

**Table 1:** Physicochemical determinations performed.

Parameter	Determination Method
pH	The multifunctional analyser CX-461 (Elmetron, Zabrze, Poland).
Conductivity	
TOC	TOC-L series analyser (Shimadzu, Kioto, Prefektura Kioto, Japan).
COD	Test Method—UV–Vis Spectroquant^®^ Prove 100 (Merck, Kenilworth, NJ, USA). (Measuring ranges: NO_3_—0.2–20 mgN-NO_3_/L, PO_4_—0.5–30 mgP-PO_4_/L), colour—0–2500 mgPt/L, COD—10–150 mgO_2_/L, Cu—0.02–6.0 mgCu/L, Ni—0.02–6.0 mgNi/L, Pb—0.01–5.0 mgPb/L, Zn—0.2–5.0 mgZn/L.
Nitrate nitrogen	
Phosphate phosphorus	
Colour	
Copper	
Nickel	
Zinc	
Lead	

**Table 2:** Structure parameters of pristine and functionalized mesoporous silica carriers.

Sample	S_BET_ [m^2^/g]	Vp_N2_ [cm^3^/g]	dp [nm]	dw [nm]
MCF	480	2.1	24	15
MCF-NH_2_	300	1.5	19.7	13.0
MCF-SH	370	1.8	23.1	14.3
MCF-NH_2_/SH	380	1.9	22.1	14.9

S_BET_—specific surface area determined by low-temperature nitrogen adsorption and using the Brunauer–Emmett–Teller (BET) method; Vp_N2_—pore volume determined by low-temperature nitrogen adsorption and using the Barrett–Joyne–Halenda (BJH) method; dp—pore diameter; dw—window diameter.

**Table 3:** Characteristics of rainwater.

Parameter	Unit	Rainwater	Polish Regulation [47], Journal of Laws 2019, Item 1311
pH	-	6.7	6.5–9.0
Conductivity	mS/cm	5.78	-
Colour	mgPt/L	21	-
TOC	mgC/L	2.64	30.0
COD	mgO_2_/L	63	125.0
Hardness	mval/L	3.2	-
N-NO_3_^−^	mg/L	3.3	30
P-PO_4_^3−^	mg/L	0.0	1.0
Zn	mg/L	4.7	2.0
Cu	mg/L	0.00	0.5
Ni	mg/L	0.07	0.5
Pb	mg/L	0.00	0.5

**Table 4:** Organic compounds identified in rainwater.

Compounds	Rt [min]	CAS Number	Molar Mass [g/mol]
Neodecanoic acid	14.977	26896-20-8	214.34
Benzothiazole	15.058	95-16-9	135.19
2-Hydroxyhexadecyl butanoate	15.346	145590-21-9	314.5
Hexanoic acid, 2-ethyl-, methyl ester	15.496	7425-23-2	158.24
Pentanoic acid, 2-acetyl-4-methyl-, methyl ester	16.059	38629-82-0	186.23
Phenol, 2,6-dimethoxy-	18.442	91-17-8	182.21
(1R,2R,3S,5R)-(-)-2,3-Pinanediol	18.573	4770-94-9	166.22
2-Piperidinone, N-[4-bromo-n-butyl]-	19.043	27761-74-8	242.10
Benzyl oleate	21.445	3687-45-4	316.46
5-tert-Butylpyrogallol	21.814	96-26-4	214.24
2,4-Di-tert-butylphenol	22.652	96-76-4	206.32
Hexadecane	24.635	544-76-3	226.43
Dodecane, 2,6,10-trimethyl-	24.86	3341-81-3	224.43
Heptacosane	27.813	629-80-1	396.86
Tetradecane, 2,6,10-trimethyl-	28.075	594-82-1	226.43
Octadecane	29.289	593-45-3	254.51
Diisobutyl phthalate	30.553	84-69-5	278.34
Dibutyl phthalate	32.454	84-74-2	278.34

**Table 5:** Parameters of the Freundlich, Langmuir, and Dubinin–Radushkevich equations, and the correlation coefficients for the adsorption of zinc and colour on the sorbents.

Adsorbent	Langmuir			Freundlich			Dubinin–Radushkevich	
	Q_m_ (mg/g)	K_L_ (L/mg)	R^2^ (-)	K_F_ ((mg/g) L/mg)^n^)	n (-)	R^2^ (-)	E (J/mol)	R^2^ (-)
Zinc	52.36	0.06	0.91	3.03	0.88	0.93	1054.9	0.917
Colour	47.62	0.10	0.64	2.10	0.34	0.83	1825.74	0.836

**Table 6:** Parameters of the pseudo-first- and second-order kinetic models for the adsorption of zinc and colour.

Adsorbate	Pseudo-First-Order Equation Parameters			Pseudo-Second-Order Equation Parameters		
	K_1_ (1/min)	Qe (mg/g)	R^2^	K_2_ (g/(mg·min))	Qe (mg/g)	R^2^
Zinc	0.0205	13.82	0.989	0.01	4.89	0.997
Colour	0.001	34.94	0.389	0.072	83.3	1

## Data Availability

Data are contained within the article.

## References

[1] Gabr M.E., El Shorbagy A.M., Faheem H.B. (2023). Assessment of Stormwater Quality in the Context of Traffic Congestion: A Case Study in Egypt. Sustainability.

[2] Generowicz A., Gronba-Chyła A., Kulczycka J., Harazin P., Gaska K., Ciuła J., Ocłoń P. (2023). Life Cycle Assessment for the environmental impact assessment of a city’ cleaning system. The case of Cracow (Poland). J. Clean. Prod..

[3] Emmanuel E., Balthazard-Accou K., Osnick J. (2009). Impact of Urban Wastewater on Biodiversity of Aquatic Ecosystems. Environmental Management, Sustainable Development and Human Health.

[4] Gronba-Chyła A., Generowicz A., Kwaśnicki P., Cycoń D., Kwaśny J., Grąz K., Gaska K., Ciuła J. (2022). Determining the Effectiveness of Street Cleaning with the Use of Decision Analysis and Research on the Reduction in Chloride in Waste. Energies.

[5] Göbel P., Dierkes C., Coldewey W.G. (2007). Storm water runoff concentration matrix for urban areas. J. Contam. Hydrol..

[6] Zhang D.-Q., Gersberg R., Ng J., Tan S. (2015). Conventional and decentralized urban stormwater management: A comparison through case studies of Singapore and Berlin, Germany. Urban Water J..

[7] Yang Y., Zhang X., Jiang J., Han J., Li W., Li X., Leung K.M.Y., Snyder S.A., Alvarez P.J.J. (2022). Which Micropollutants in Water Environments Deserve More Attention Globally?. Environ. Sci. Technol..

[8] Kumar A., Deepika, Tyagi D., Tarkeshwar, Kapinder, Bhadouria R., Tripathi S., Singh P., Singh R., Singh H.P. (2024). Organic Micropollutants and Their Effects on the Environment and Human Health. Organic Micropollutants in Aquatic and Terrestrial Environments.

[9] Villagómez-Márquez N., Abrell L., Foley T., Ramírez-Andreotta M.D. (2023). Organic micropollutants measured in roof-harvested rainwater from rural and urban environmental justice communities in Arizona. Sci. Total Environ..

[10] Gasperi J., Sebastian C., Ruban V., Delamain M., Percot S., Wiest L., Mirande C., Caupos E., Demare D., Kessoo M.D.K. (2014). Micropollutants in urban stormwater: Occurrence, concentrations, and atmospheric contributions for a wide range of contaminants in three French catchments. Environ. Sci. Pollut. Res..

[11] Lee T.H.Y., Srinuansom K., Snyder S.A., Ziegler A.D. (2023). Atmosphere-Transported Emerging and Persistent Contaminants (EPCs) in Rainfall and Throughfall: Insights from a Rural Site in Northern Thailand. Atmosphere.

[12] Fuchte H.E., Beck N., Bieg E., Bayer V.J., Achten C., Krauss M., Schäffer A., Smith K.E.C. (2022). A look down the drain: Identification of dissolved and particle bound organic pollutants in urban runoff waters and sediments. Environ. Pollut..

[13] Iroegbulema I.U., Egereonu U.U., Ogukwea C.E., Egereonub J.C., Okorocand N.J., Nwoko C.I.A. (2023). Assessment of Heavy Metals in Rainwater from Metropolis and Suburbs, Lagos State, Nigeria. Int. J. Environ. Clim. Chang..

[14] Başak B., Alagha O. (2010). Trace metals solubility in rainwater: Evaluation of rainwater quality at a watershed area, Istanbul. Env. Monit. Assess..

[15] Cheng M.-C., You C.-F., Lin F.-J., Huang K.-F., Chung C.-H. (2011). Sources of Cu, Zn, Cd and Pb in rainwater at a subtropical islet offshore northern Taiwan. Atmos. Environ..

[16] Sharma P., Rai V. (2018). Assessment of rain water chemistry in the Lucknow metropolitan city. Appl. Water Sci..

[17] Hatt B.E., Deletic A., Fletcher T.D. (2006). Integrated treatment and recycling of stormwater: A review of Australian practice. J. Environ. Manag..

[18] Ciuła J., Gaska K., Siedlarz D., Koval V. (2019). Management of sewage sludge energy use with the application of bi-functional bioreactor as an element of pure production in industry. E3S Web Conf..

[19] Ewis D., Ba-Abbad M.M., Benamor A., El-Naas M.H. (2022). Adsorption of organic water pollutants by clays and clay minerals composites: A comprehensive review. Appl. Clay Sci..

[20] Azha S.F., Shahadat M., Ismail S., Ali S.W., Ahammad S.Z. (2021). Prospect of clay-based flexible adsorbent coatings as cleaner production technique in wastewater treatment, challenges, and issues: A review. J. Taiwan Inst. Chem. Eng..

[21] Tehrani N.H.M.H., Ardjmand M., Bazmi M., Rashidi A., Zadeh M.R.M. (2023). Polydopamine-modified mesoporous silica materials as a novel adsorbent for superior CO_2_ adsorption: Experimental and DFT study. J. Environ. Chem. Eng..

[22] Addy M., Losey B., Mohseni R., Zlotnikov E., Vasiliev A. (2012). Adsorption of heavy metal ions on mesoporous silica-modified montmorillonite containing a grafted chelate ligand. Appl. Clay Sci..

[23] Ciuła J., Kowalski S., Wiewiórska I. (2023). Pollution Indicator of a Megawatt Hour Produced in Cogeneration—The Efficiency of Biogas Purification Process as an Energy Source for Wastewater Treatment Plants. J. Ecol. Eng..

[24] Ueta I., Hayashibe M., Sumiya K., Ariizumi Y., Fujimura K., Saito Y. (2023). Polydimethylsiloxane-coated macroporous silica adsorbent in thermal desorption gas chromatography. J. Chromatogr. Open.

[25] Li H., Chen X., Shen D., Wu F., Pleixats R., Pan J. (2021). Functionalized silica nanoparticles: Classification, synthetic approaches and recent advances in adsorption applications. Nanoscale.

[26] Wieszczycka K., Wojciechowska I., Filipowiak K., Buchwald T., Nowicki M., Dudzinska P., Strzemiecka B., Voelkel A. (2022). Novel iminepyridinium-modified silicas as super-adsorbents for metals ions. Appl. Surf. Sci..

[27] Angotzi M.S., Mameli V., Cara C., Borchert K.B.L., Steinbach C., Boldt R., Schwarz D., Canna C. (2021). Meso- and macroporous silica-based arsenic adsorbents: Effect of pore size, nature of the active phase, and silicon release. Nanoscale Adv..

[28] Wang P., Du M., Zhu H., Bao S., Yang T., Zou M. (2015). Structure regulation of silica nanotubes and their adsorption behaviors for heavy metal ions: pH effect, kinetics, isotherms and mechanism. J. Hazard. Mat..

[29] Chaabane L., Nikolantonaki M., Weber G., Bezverkhyy I., Chassagnon R., Assifaoui A., Bouyer F. (2023). Functionalization of SBA-15 mesoporous silica for highly efficient adsorption of glutathione: Characterization and modeling studies. J. Taiwan Inst. Chem. Eng..

[30] Antony J., Gonzalez S.V., Bandyopadhyay S., Yang J., Rønning M. (2023). Silica-modified bismutite nanoparticles for enhanced adsorption and faster solar photocatalytic degradation of methylene blue. Catal. Today.

[31] Cheng L., Sun X., Li Q., Yang J., Wang R., Sun X., Wei L. (2024). Construction of C8/threonine-functionalized mesoporous silica aerogel based on mixed-mode and its adsorption properties for both anionic and cationic dyes. Powder Technol..

[32] Shan W., Zhang Y., Shu Y., Zhang D., Xing C., Xiong Y. (2021). Enhanced adsorption and separation of Re(VII) using organic-inorganic hybrid silica adsorbent. Microporous Mesoporous Mater..

[33] Da’na E. (2017). Adsorption of heavy metals on functionalized-mesoporous silica: A review. Microporous Mesoporous Mater..

[34] Cao Y., Malekshah R.E., Heidari Z., Pelalak R., Marjani A., Shirazian S. (2021). Molecular dynamic simulations and quantum chemical calculations of adsorption process using amino-functionalized silica. J. Mol. Liq..

[35] Lamy-Mendes A., Torres R.B., Vareda J.P., Lopes D., Ferreira M., Valente V., Girão A.V., Valente A.J.M., Durães L. (2019). Amine Modification of Silica Aerogels/Xerogels for Removal of Relevant Environmental Pollutants. Molecules.

[36] Kudlek E. (2018). Decomposition of contaminants of emerging concern in advanced oxidation processes. Water.

[37] Jarzębski A.B., Szymańska K., Bryjak J., Mrowiec-Białoń J. (2007). Covalent immobilization of trypsin on to siliceous mesostructured cellular foams to obtain effective biocatalysts. Catal. Today.

[38] Szymańska K., Bryjak J., Jarzębski A.B. (2009). Immobilization of Invertase on Mesoporous Silicas to Obtain Hyper Active Biocatalysts. Top. Catal..

[39] Kamińska G., Bohdziewicz J. (2016). Potential of various materials for adsorption of micropollutants from wastewater. Environ. Prot. Eng..

[40] Marszałek A., Kamińska G., Fathy Abdel Salam N. (2022). Simultaneous adsorption of organic and inorganic micropollutants from rainwater by bentonite and bentonite-carbon nanotubes composites. J. Water Process Eng..

[41] Maury S., Buisson P., Pierre A.C. (2001). Porous texture modification of silica aerogels in liquid media and its effect on the activity of a lipase. Langmuir.

[42] Zhang X., Guan R.-F., Wu D.-Q., Chan K.-Y. (2005). Enzyme immobilization on amino-functionalized mesostructured cellular foam surfaces, characterization and catalytic properties. J. Mol. Catal. B Enzym..

[43] Karbowiak T., Saada M.A., Rigolet S., Ballandras A., Weber G., Bezverkhyy I., Soulard M., Patarin J., Bellat J.-P. (2010). New insights in the formation of silanol defects in silicalite-1 by water intrusion under high pressure. Phys. Chem. Chem. Phys..

[44] Chrzanowska A., Derylo-Marczewska A., Wasilewska M. (2020). Mesocellular silica foams (MCFs) with tunable pore size as a support for lysozyme immobilization: Adsorption equilibrium and kinetics, biocomposite properties. Int. J. Mol. Sci..

[45] Ciemięga A., Maresz K., Mrowiec-Białoń J. (2018). Meervein-Ponndorf-Vereley reduction of carbonyl compounds in monolithic siliceous microreactors doped with Lewis acid centres. Appl. Catal. A Gen..

[46] Szymańska K., Bryjak J., Mrowiec-Białoń J., Jarzębski A.B. (2007). Application and properties of siliceous mesostructured cellular foams as enzymes carriers to obtain efficient biocatalysts. Microporous Mesoporous Mater..

[47] Regulation of the Minister of Maritime Economy and Inland Navigation. Journal of Laws 2019, item 1311. https://isap.sejm.gov.pl/isap.nsf/DocDetails.xsp?id=WDU20190001311.

[48] Sieber G., Beisser D., Rothenberger J., Shah M., Schumann M., Sures B., Boenigk J. (2022). Microbial community shifts induced by plastic and zinc as substitutes of tire abrasion. Sci. Rep..

[49] Wicke D., Matzinger A., Sonnenberg H., Caradot N., Schubert R.-L., Dick R., Heinzmann B., Dünnbier U., von Seggern D., Rouault P. (2021). Micropollutants in Urban Stormwater Runoff of Different Land Uses. Water.

[50] Jakubowicz P., Fitobór K., Gajewska M., Drewnowska M. (2022). Detection and Removal of Priority Substances and Emerging Pollutants from Stormwater: Case Study of the Kołobrzeska Collector, Gdańsk, Poland. Sustainability.

[51] Westbrook C.K., Pitz W.J., Herbinet O., Curran H.J., Silke E.J. (2009). A comprehensive detailed chemical kinetic reaction mechanism for combustion of n-alkane hydrocarbons from n-octane to n-hexadecane. Combust. Flame.

[52] Feltracco M., Mazzi G., Barbaro E., Rosso B., Sambo F., Biondi S., Gambaro A. (2023). Occurrence and phase distribution of benzothiazoles in untreated highway stormwater runoff and road dust. Environ. Sci. Pollut. Res..

[53] Struk-Sokołowska J., Gwoździej-Mazur J., Jurczyk Ł., Jadwiszczak P., Kotowska U., Piekutin J., Canales F.A., Kaźmierczak B. (2022). Environmental risk assessment of low molecule benzotriazoles in urban road rainwaters in Poland. Sci. Total Environ..

[54] Men C., Liu R., Xu F., Wang Q., Guo L., Shen Z. (2018). Pollution characteristics, risk assessment, and source apportionment of heavy metals in road dust in Beijing, China. Sci. Total Environ..

[55] Kim L.-H., Kayhanian M., Zoh K.-D., Stenstrom M.K. (2005). Modeling of highway stormwater runoff. Sci. Total Environ..

[56] Jensen D.M.R., Thomsen A.T.H., Larsen T., Egemose S., Mikkelsen P.S. (2020). From EU Directives to Local Stormwater Discharge Permits: A Study of Regulatory Uncertainty and Practice Gaps in Denmark. Sustainability.

[57] Novaes C.A.F.O., Marques R.C. (2022). Regulation of urban stormwater management is not a matter of choice, but performance. Water Policy.

[58] Bichai F., Ashbolt N. (2017). Public health and water quality management in low-exposure stormwater schemes: A critical review of regulatory frameworks and path forward. Sustain. Cities Soc..

[59] Hafezian S.M., Biparva P., Bekhradnia A., Azizi S.N. (2021). Amine and thiol functionalization of SBA-15 nanoparticles for highly efficient adsorption of sulforaphane. Adv. Powder Technol..

[60] Xie Y., Lyu S., Zhang Y., Cai C. (2022). Adsorption and Degradation of Volatile Organic Compounds by Metal–Organic Frameworks (MOFs): A Review. Materials.

[61] Pourhakkak P., Taghizadeh M., Taghizadeh A., Ghaedi M., Ghaedi M. (2021). Adsorbent. Adsorption: Fundamental Processes and Applications, Interface Science and Technology.

[62] Nakanishi K., Tomita M., Kato K. (2015). Synthesis of amino-functionalized mesoporous silica sheets and their application for metal ion capture. J. Asian Ceram. Soc..

[63] Melnyk I.V., Tomina V.V., Stolyarchuk N.V., Seisenbaeva G.A., Kessler V.G. (2021). Organic dyes (acid red, fluorescein, methylene blue) and copper(II) adsorption on amino silica spherical particles with tailored surface hydrophobicity and porosity. J. Mol. Liq..

[64] Li J., Miao X., Hao Y., Zhao J., Sun X., Wang L. (2008). Synthesis, amino-functionalization of mesoporous silica and its adsorption of Cr(VI). J. Colloid Interface Sci..

[65] Wang J., Zheng S., Liu J., Xu Z. (2010). Tannic acid adsorption on amino-functionalized magnetic mesoporous silica. Chem. Eng. J..

[66] Aguado J., Arsuaga J.M., Arencibia A., Lindo M., Gascón V. (2009). Aqueous heavy metals removal by adsorption on amine-functionalized mesoporous silica. J. Hazard. Mater..

[67] Huynh J., Palacio R., Allavena A., Gallard H., Descostes M., Mamède A.-S., Royer S., Tertre E., Batonneau-Gener I. (2021). Selective adsorption of U(VI) from real mine water using an NH_2_-functionalized silica packed column. Chem. Eng. J..

[68] Jayalath S., Larsen S.C., Grassian V.H. (2018). Surface adsorption of Nordic aquatic fulvic acid on amine-functionalized and non-functionalized mesoporous silica nanoparticles. Environ. Sci. Nano.

[69] Ebrahimi-Gatkash M., Younesi H., Shahbazi A., Heidari A. (2017). Amino-functionalized mesoporous MCM-41 silica as an efficient adsorbent for water treatment: Batch and fixed-bed column adsorption of the nitrate anion. Appl. Water Sci..

[70] Nayab S., Farrukh A., Oluz Z., Tuncel E., Tariq S.R., Rahman H., Kirchhoff K., Duran H., Yameen B. (2014). Design and Fabrication of Branched Polyamine Functionalized Mesoporous Silica: An Efficient Absorbent for Water Remediation. ACS Appl. Mater. Interfaces.

[71] Flores D., Almeida C.M.R., Gomes C.R., Balula S.S., Granadeiro C.M. (2023). Tailoring of Mesoporous Silica-Based Materials for Enhanced Water Pollutants Removal. Molecules.

[72] Brigante M., Pecini E., Avena M. (2016). Magnetic mesoporous silica for water remediation: Synthesis, characterization and application as adsorbent of molecules and ions of environmental concern. Microporous Mesoporous Mater..

[73] Zhao H., Nagy K.L., Waples J.S., Vance G.F. (2000). Surfactant-Templated Mesoporous Silicate Materials as Sorbents for Organic Pollutants in Water. Environ. Sci. Technol..

[74] Wang T., Li Y., Wu J., Hu Y. (2024). Pivotal role of pH value in the preparation of mesoporous silica with high surface area for toluene adsorption. Mater. Lett..

[75] Mobasherpour I., Salahi E., Ebrahimi M. (2014). Thermodynamics and kinetics of adsorption of Cu(II) from aqueous solutions onto multi-walled carbon nanotubes. J. Saudi Chem. Soc..

[76] Makara A., Kowalski Z., Sówka I. (2016). Possibility to eliminate emission of odor from pig manure using AMAK filtration method. Desalin. Water Treat..

[77] Zhang Y., Jia S., Yuan X., Ding L., Ai T., Yuan K., Wang W., Magagnin L., Jiang Z. (2024). High-efficiency removal of Pb (II) and Cu (II) by amidoxime functionalized silica aerogels: Preparation, adsorption mechanisms and environmental impacts analysis. Sep. Purif. Technol..

[78] Wang H., Kang J., Liu H., Qu J. (2009). Preparation of organically functionalized silica gel as adsorbent for copper ion adsorption. J. Environ. Sci..

[79] Zhang P., He M., Teng W., Li F., Qiu X., Li K., Wang H. (2023). Ordered mesoporous materials for water pollution treatment: Adsorption and catalysis. Green Energy Environ..

